# Microbial Biocontrol in the Agri-Food Industry

**DOI:** 10.3390/microorganisms11030552

**Published:** 2023-02-21

**Authors:** Maurizio Ciani

**Affiliations:** Department of Life and Environmental Sciences, Polytechnic University of Marche, Via Brecce Bianche, 60131 Ancona, Italy; m.ciani@univpm.it; Tel.: +39-071-220-4987

**Keywords:** antimicrobial compounds, food industry, spoilage microorganisms, killer yeasts, essential oils, control of plant pathogens

## Abstract

In recent years there has been a growing interest in the use of natural antimicrobial compounds to limit or avoid the use of chemical antimicrobials. Natural antimicrobial compounds can come from plants (essential oils) or from microorganisms (bacteriocins, mycocines, and active peptides). Despite a wide range of possible applications, their exploitation at the industrial level is still limited and needs to be investigated. The actual and possible applications of natural antimicrobial compounds in agri-food are a growing research field. In addition to the use of antimicrobial compounds, microorganisms themselves can be used in the control of spoilage microorganisms along the entire production chain of the agri-food industry. Likewise, the papers collected in this Special Issue indicate the fast development of novelties in this research field.

## 1. Introduction

Natural antimicrobial compounds are a wide range of compounds of various natural origins that possess antimicrobial activity against undesirable microorganisms or pathogens for plants, animals, and humans. Some microorganisms, especially yeasts and molds, can be directly used as biocontrol agents during the field cultivation of crops or during their transformation processes. On the other hand, natural compounds with antimicrobial activity can be used as concentrated extracts in addition to semi-purified or activated preparations. This Special Issue collected research papers on recent developments within this research topic.

## 2. Natural Antimicrobial Compounds

A microbiological approach based on the selection of bio-protective strains can be a useful tool for controlling undesired microorganisms of plant diseases [1,2,3]. Some studies have investigated the biocontrol action against the gray mold disease agent *Botrytis cinerea* in vineyard by yeasts [4] as well as in fruit and vegetable spoilage phenomena by lactic acid bacteria in postharvest [5]. Promising results have indicated that yeast strains belonging to *Metschnikowia pulcherrima* and *Aureobasidium pullulans* exhibited the ability to contain the development of *B. cinerea*, while among lactic acid bacteria some strains belonging to *Lactiplantibacillus plantarum* species exerted a strong antagonism against *B. cinerea*. The antimicrobial activity of essential oils from indigenous Iranian plants was assayed against multidrug-resistant *Escherichia coli* strains [6]. The results indicated that the essential oil of *Zataria multiflora* can be used as a practical and alternative antibacterial strategy to inhibit the growth of multidrug-resistant *E. coli* strains. Leaf extracts of *Moringa oleifera* Lam., a tropical plant, have antibacterial action against both Gram-negative and Gram-positive bacteria. These extracts, containing many bioactive compounds, such as flavonoids, phenolic acids, alkaloids, isothiocyanates, tannins, and saponins, showed bactericidal effects against *Xanthomonas campestris* pv. *campestris* (Xcc), a Gram-negative bacterium that causes black rot in crucifers [7].

Among biopolymers with antimicrobial activity, chitin and its derivatives are promising for use in the management of plant disease. In this Special Issue, the antimicrobial activities of chitin and chitosan extracted from black soldier fly (BSF) pupal exuviae were evaluated. A paper described the first comparative analysis of the chemical and biological extraction of chitin and chitosan from BSF pupal exuviae, a byproduct that could be a valuable byproduct of insect-farming enterprises [8]. Another paper reported the antibacterial activity of BSF chitosan against *Ralstonia solanacearum* [9]; the results reported the significant inhibition of *R. solanacearum* by chitosan from BSF.

Mycocins are a group of natural antimicrobials of microbial origin with application developments. A mycocin from *Wickerhamomyces anomalus*, named WA18, with a broad spectrum of activity, was purified and widely investigated [10]. The practical application in winemaking conditions of WA18 partially purified crude extract revealed stable antimicrobial activity that counteracted *Brettanomyces bruxellensis* and controlled the production of undesired ethyl phenols. The use of antimicrobial compounds of different origins may be considered a very promising approach to reduce the use of chemical compounds in agriculture and food processing for the more sustainable production and control of microbial decay [11,12]. Among them, the killer yeasts belonging to *Kluyveromyces wickerhamii*, *Wickerhamomyces anomalus*, and *Pichia membranifaciens* species represent excellent examples extensively studied for their efficacy toward different sensitive spoilage yeasts in the winemaking field.

## Data Availability

Not applicable.
